# Rheumatoid Arthritis-Associated Interstitial Lung Disease Presenting With Raynaud's Phenomenon: A Case Report

**DOI:** 10.7759/cureus.107697

**Published:** 2026-04-25

**Authors:** Prashant Ghule, Anil Sontakke, Saood Ali, Sagar Kolte, Somesh Mashalkar

**Affiliations:** 1 Respiratory Medicine, N.K.P. Salve Institute of Medical Sciences and Research Centre and Lata Mangeshkar Hospital (NKPSIMS and LMH), Nagpur, IND

**Keywords:** autoimmune lung disease, high-resolution ct, interstitial lung disease, raynaud's phenomenon, rheumatoid arthritis, usual interstitial pneumonia

## Abstract

Rheumatoid arthritis-associated interstitial lung disease (RA-ILD) is a recognized extra-articular manifestation of rheumatoid arthritis. Pulmonary involvement may occasionally precede overt articular disease, creating diagnostic challenges. A patient presented with breathlessness for 2-3 months and joint pain of similar duration, along with bluish discoloration of the left middle finger, suggestive of Raynaud’s phenomenon. Respiratory examination revealed bilateral inspiratory crepitations in the infrascapular and infra-axillary areas. Rheumatoid factor was positive, while antinuclear antibody was negative. High-resolution computed tomography (HRCT) of the thorax demonstrated microcystic honeycombing, basal subpleural fibrosis, and traction bronchiectasis, consistent with a usual interstitial pneumonia (UIP) pattern. This case highlights ILD as a potential early manifestation of rheumatoid arthritis and emphasizes the importance of autoimmune evaluation in patients presenting with fibrotic ILD and systemic features such as Raynaud’s phenomenon.

## Introduction

Rheumatoid arthritis is a chronic, systemic autoimmune disorder characterized primarily by persistent synovial inflammation, leading to progressive joint destruction and functional disability. In addition to articular involvement, rheumatoid arthritis frequently manifests with extra-articular complications affecting multiple organ systems, including the lungs, cardiovascular system, skin, and hematologic system. Among these, pulmonary involvement is one of the most clinically significant manifestations and contributes substantially to morbidity and mortality in patients with rheumatoid arthritis [1]. Interstitial lung disease (ILD) represents one of the most important pulmonary complications associated with rheumatoid arthritis. The reported prevalence of rheumatoid arthritis-associated interstitial lung disease (RA-ILD) ranges from 20% to 60%, of which approximately 35%-45% of patients show progression of CT abnormalities. Patients with RA-ILD commonly present with progressive exertional dyspnea, persistent dry cough, and reduced exercise tolerance [2]. In certain cases, pulmonary manifestations may precede the onset of clinically apparent joint disease, which may delay diagnosis. High-resolution computed tomography (HRCT) plays a crucial role in the diagnosis and evaluation of RA-ILD [3]. Several radiological patterns have been described, among which usual interstitial pneumonia (UIP) is the most frequently encountered. The UIP pattern is characterized by basal and subpleural predominance of fibrosis, honeycombing, reticular opacities, and traction bronchiectasis. This pattern is clinically important because it is associated with disease progression and poorer outcomes, similar to idiopathic pulmonary fibrosis in many cases [4]. Raynaud’s phenomenon, characterized by episodic vasospasm of the digital arteries leading to color changes in the fingers or toes, is commonly associated with connective tissue diseases such as systemic sclerosis and mixed connective tissue disease. Although less frequently reported in rheumatoid arthritis, its presence may indicate systemic vascular involvement and heightened autoimmune activity [5]. Early identification of pulmonary involvement in patients with rheumatoid arthritis is essential, as delayed diagnosis may lead to progressive fibrotic lung damage and respiratory impairment. Recognition of underlying autoimmune disease in patients presenting with ILD also has important therapeutic implications, as treatment strategies may differ from those used in idiopathic interstitial pneumonias [6]. In this report, we describe a case of rheumatoid factor-positive connective tissue disease presenting with ILD showing a UIP pattern on HRCT, along with Raynaud’s phenomenon. This case highlights the importance of considering autoimmune etiologies in patients presenting with fibrotic ILD and systemic clinical features.

## Case presentation

**Figure 1:**
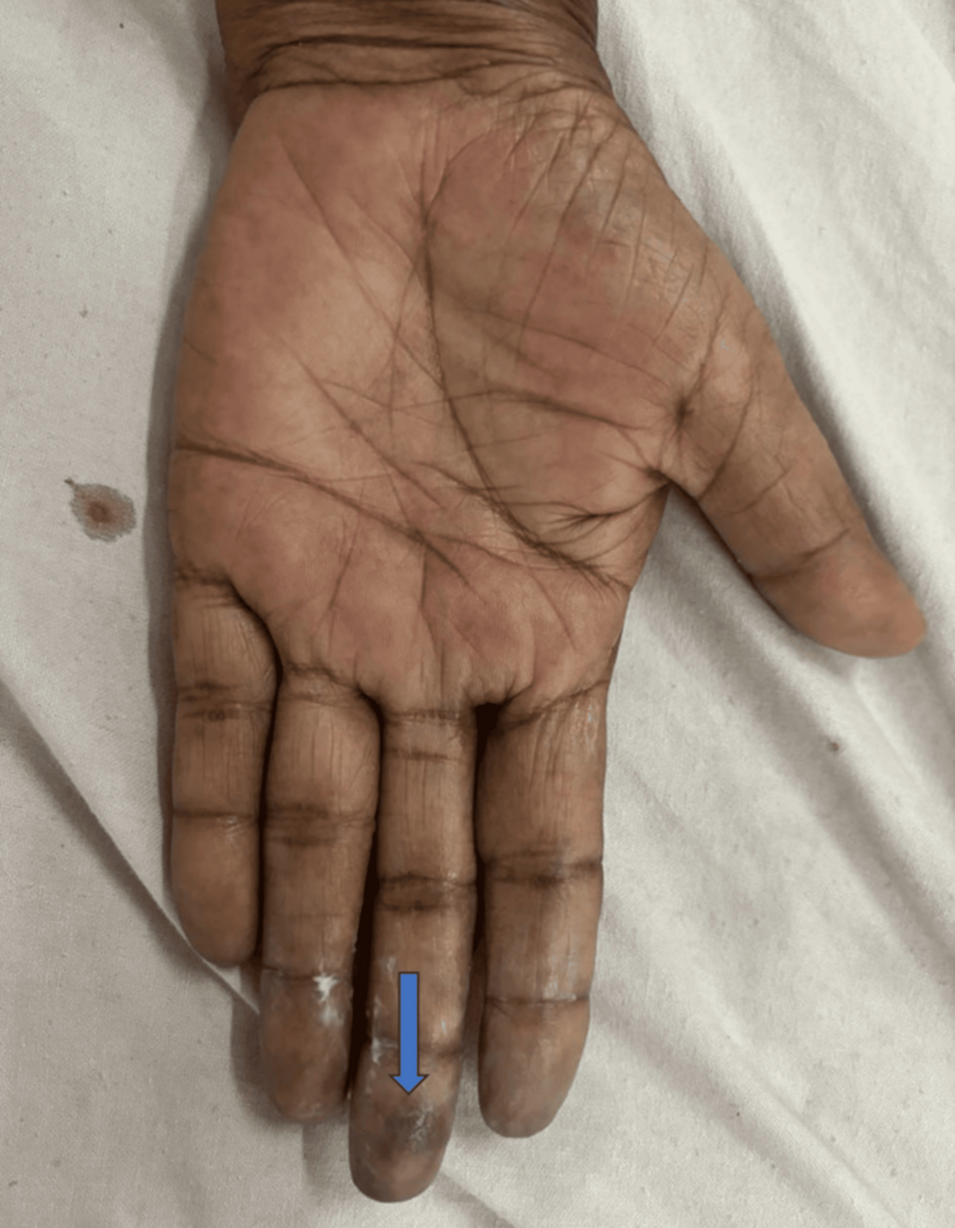
Bluish discoloration of the left middle finger (blue arrow) (palmar view)

**Figure 2:**
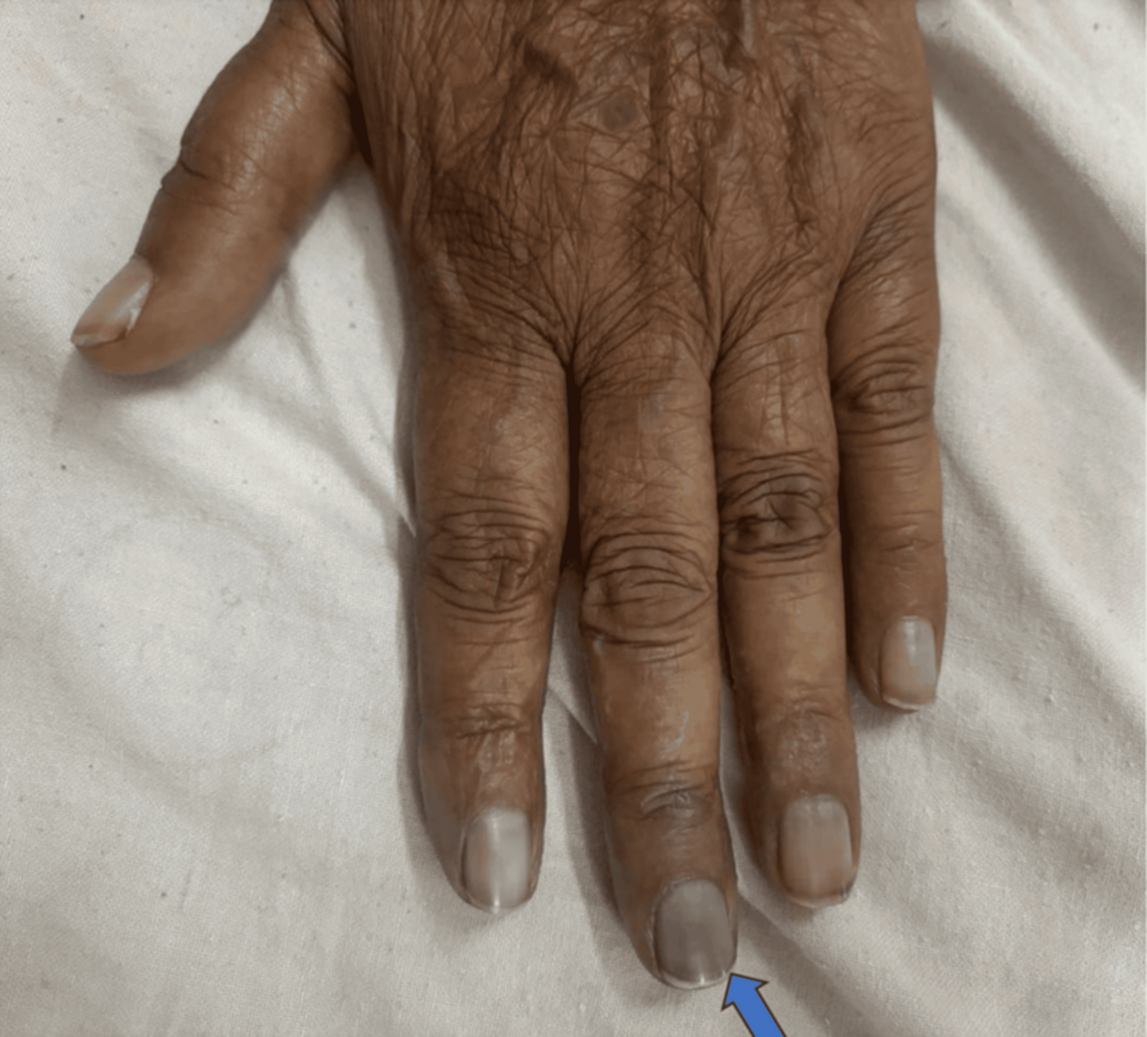
Bluish discoloration of the left middle finger (blue arrow) (dorsal view)

A 76-year-old female patient presented with progressive breathlessness on exertion for 2-3 months, along with small joint pain of similar duration. On examination, bluish discoloration of the fingers was noted (Figures 1, 2). The patient had a history of hypertension and was on regular antihypertensive therapy.

**Figure 3:**
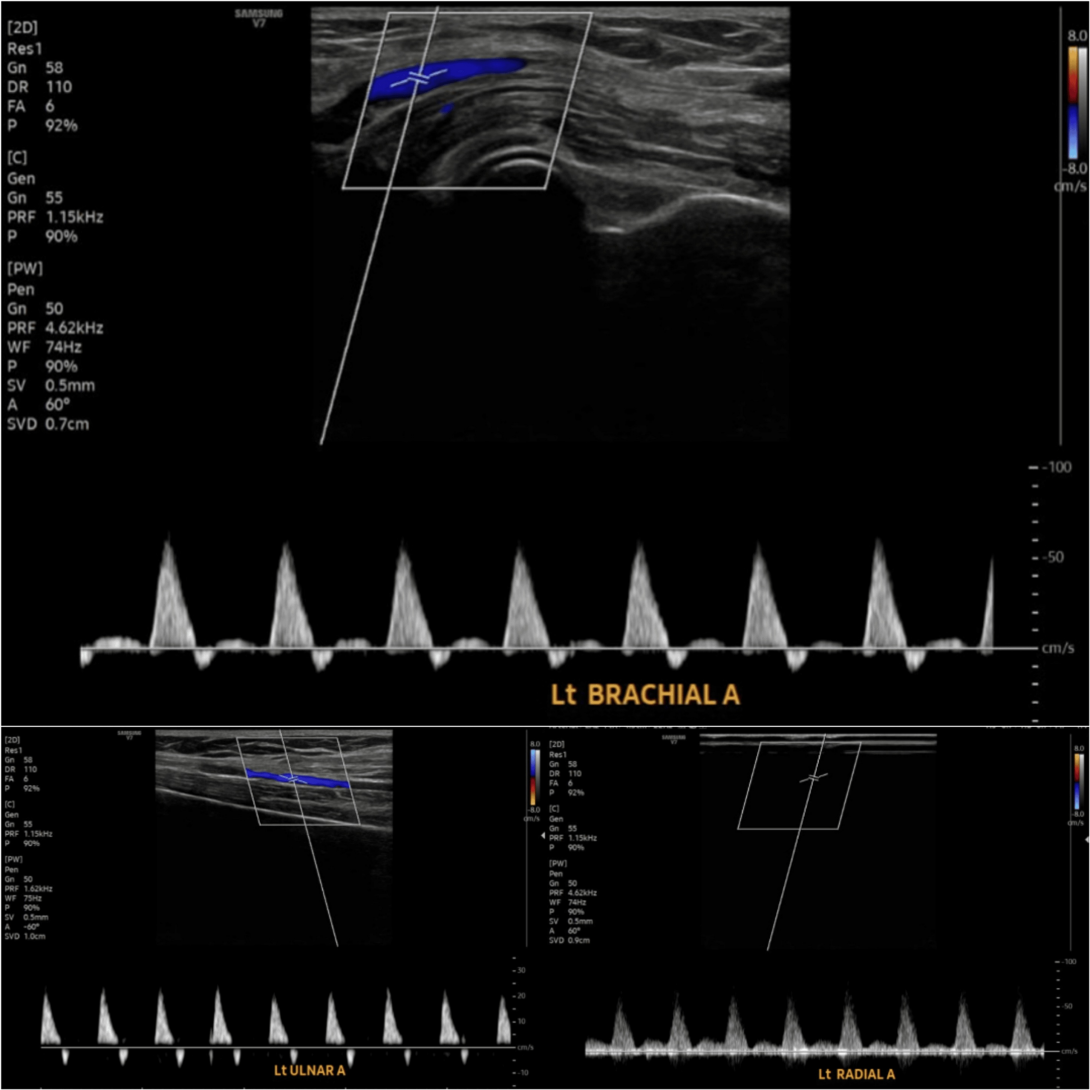
Left upper limb arterial Doppler study showing normal triphasic flow in the brachial artery and biphasic flow in the radial and ulnar arteries

Laboratory investigations showed a positive rheumatoid factor (640 IU/mL; normal <10 IU/mL) and a negative antinuclear antibody (ANA; latex agglutination method). A left upper limb arterial Doppler study demonstrated atherosclerotic vessel wall calcification, with preserved triphasic flow in the subclavian, axillary, and brachial arteries, and biphasic flow in the radial and ulnar arteries (Figure 3).

**Figure 4:**
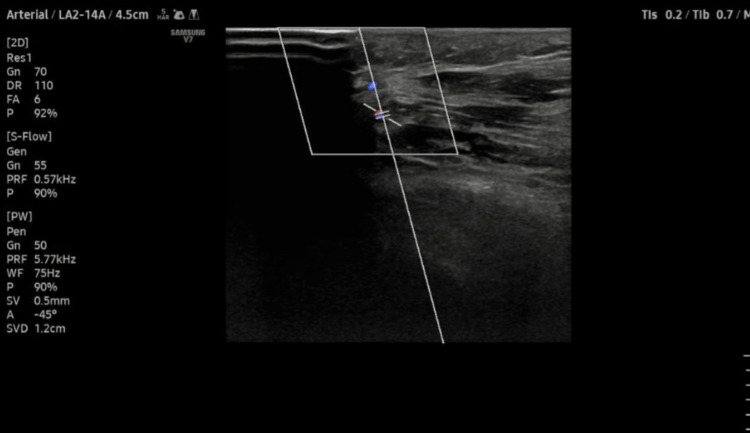
Left upper limb arterial Doppler study showing incomplete palmar arches

Allen’s test was positive, suggesting incomplete palmar arches (Figure 4).

**Figure 5:**
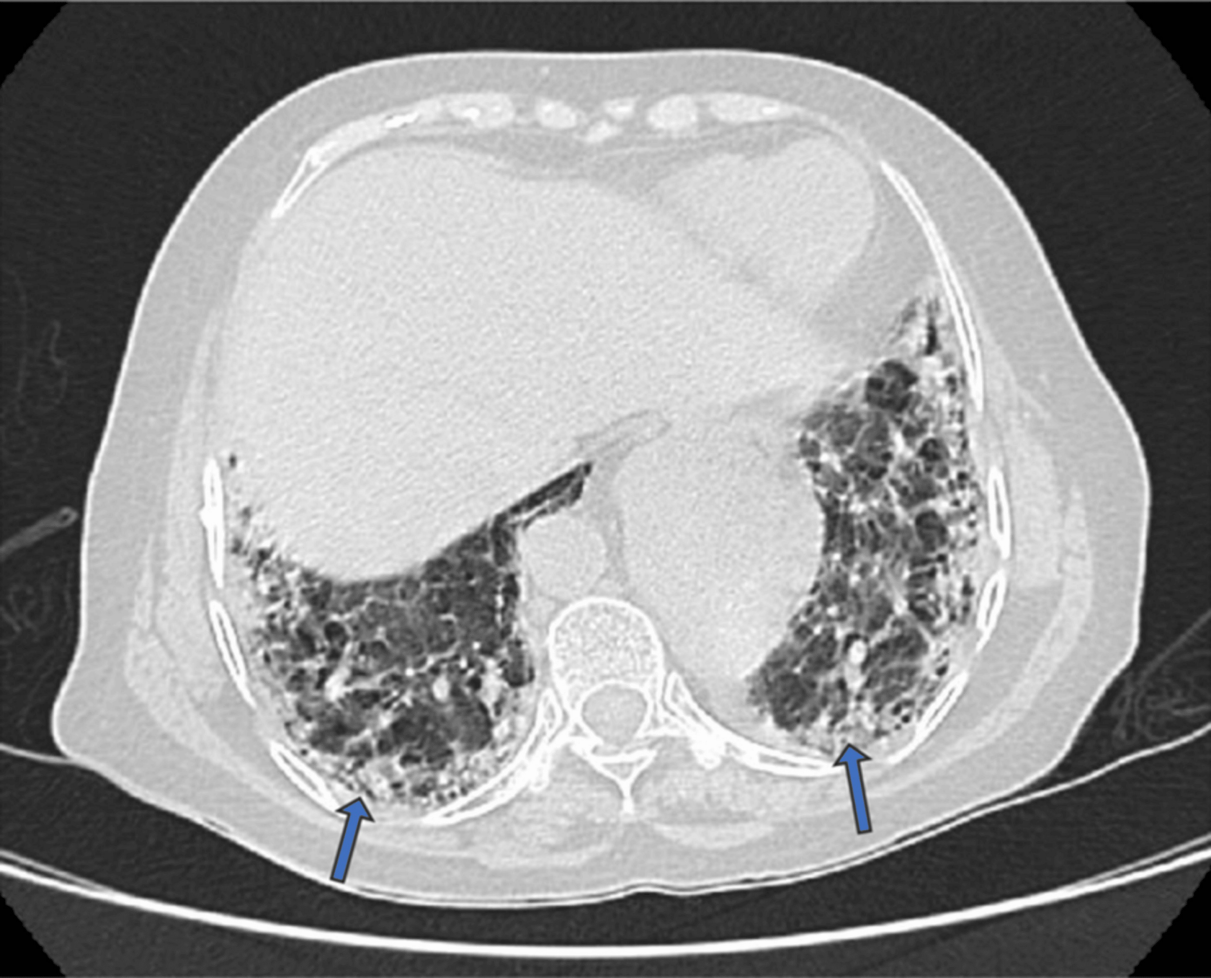
High-resolution computed tomography (HRCT) of the thorax showing microcystic honeycombing (blue arrows)

**Figure 6:**
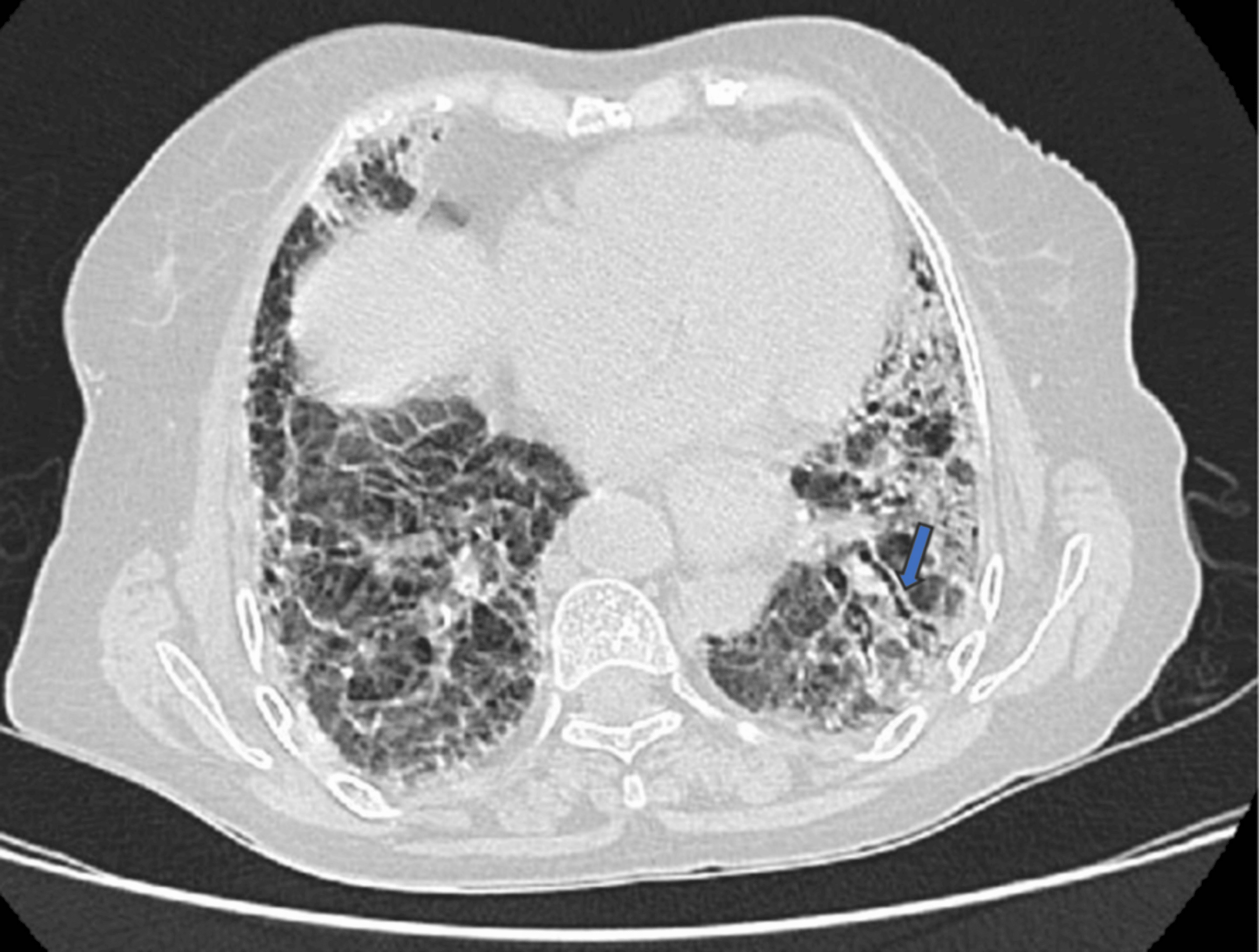
High-resolution computed tomography (HRCT) showing traction bronchiectasis changes (blue arrow)

HRCT of the thorax revealed microcystic honeycombing (Figure 5), with fibrotic changes predominantly involving the bilateral lower lobes, along with subpleural interlobular and intralobular septal thickening and traction bronchiectasis (Figure 6). The findings were consistent with a UIP pattern of ILD.

Mild cardiomegaly and a large sliding-type hiatus hernia were also noted.

## Discussion

ILD is a well-recognized extra-articular manifestation of rheumatoid arthritis and represents a major cause of morbidity and mortality among affected patients. The pathogenesis of RA-ILD is multifactorial and involves chronic immune-mediated inflammation, genetic predisposition, environmental exposures such as smoking, and abnormal repair of alveolar epithelial injury leading to progressive pulmonary fibrosis [7]. Several histopathological and radiological patterns of ILD have been described in association with rheumatoid arthritis [8]. Among these, the UIP pattern is the most commonly identified pattern on HRCT imaging. The UIP pattern typically demonstrates basal and subpleural predominant fibrosis, honeycombing, reticular abnormalities, and traction bronchiectasis [9]. The clinical presentation of RA-ILD is usually insidious, with progressive exertional dyspnea and chronic cough. Fine inspiratory crackles are often heard over the basal lung fields. In some patients, pulmonary manifestations may appear before significant joint involvement becomes clinically evident, which can complicate diagnosis [10]. The presence of rheumatoid factor positivity in this patient further supports RA-ILD [11]. Another important feature in this case was the presence of Raynaud’s phenomenon. Although Raynaud’s phenomenon is more commonly associated with systemic sclerosis and other connective tissue diseases, it may occasionally occur in rheumatoid arthritis and may reflect underlying vascular dysfunction. Early recognition of RA-ILD is important because management may include immunomodulatory therapy and close monitoring of pulmonary function. Multidisciplinary collaboration between pulmonologists, rheumatologists, and radiologists is often required for optimal management [12].

## Conclusions

This case underscores the importance of recognizing ILD as a potential early manifestation of rheumatoid arthritis, even in the absence of overt articular deformities. The presence of progressive dyspnea, rheumatoid factor positivity, and a UIP pattern on HRCT strongly supports the diagnosis of RA-ILD. Notably, the coexistence of Raynaud’s phenomenon in this patient suggests underlying systemic immune activation and vascular involvement. Pulmonary involvement may precede joint manifestations, leading to diagnostic challenges and potential delays in appropriate management. Therefore, a high index of suspicion and comprehensive autoimmune evaluation are essential in patients presenting with fibrotic ILD. Early identification is crucial, as management strategies differ from those used in idiopathic pulmonary fibrosis and may include immunomodulatory therapy. This case highlights the need for a multidisciplinary approach involving pulmonologists, rheumatologists, and radiologists to ensure accurate diagnosis and optimal treatment. Prompt recognition and targeted management may help slow disease progression and improve clinical outcomes.
